# Exploratory Study of Serum IL-22 and CD163+ Macrophages in Glioblastoma Multiforme

**DOI:** 10.3390/medicina62020253

**Published:** 2026-01-25

**Authors:** Elina Aleksandrova, Julian Ananiev, Tatyana Vlaykova, Tanya Tacheva, Hristina Petrova, Stefan Valkanov

**Affiliations:** 1 Department of Medical Chemistry and Biochemistry, Medical Faculty, Trakia University, 6000 Stara Zagora, Bulgaria; tatyana.vlaykova@trakia-uni.bg (T.V.); tanya.tacheva@trakia-uni.bg (T.T.); hristina.petrova@trakia-uni.bg (H.P.); 2 Department of General and Clinical Pathology, Forensic Medicine and Deontology, Medical Faculty, Trakia University, 6000 Stara Zagora, Bulgaria; julian.r.ananiev@trakia-uni.bg; 3 Department of Surgery, Neurosurgery, Urology and Anesthesiology, Medical Faculty, Trakia University, 6000 Stara Zagora, Bulgaria; stefan.valkanov@trakia-uni.bg

**Keywords:** glioblastoma, IL-22, CD163-positive macrophages, tumor microenvironment

## Abstract

*Background and Objectives:* Glioblastoma (GBM) is the most aggressive primary tumor of the central nervous system, characterized by high invasiveness and poor prognosis. Inflammation in the tumor microenvironment, including the presence of immunosuppressive M2-macrophages (CD163+), plays a key role in disease progression. The aim of this study was to evaluate serum levels of interleukin-22 (IL-22) in Bulgarian patients with GBM and to analyze its diagnostic role, its relationship with systemic inflammatory markers (NLR), metabolic parameters, and the infiltration of CD163+ cells. *Materials and Methods:* The study included 41 newly diagnosed patients with GBM and 46 healthy controls. Serum IL-22 levels were measured by ELISA, and the density of CD163+ cells in the tumor tissue was analyzed immunohistochemically. Statistical analysis included Mann–Whitney test, ROC analysis, binary logistic regression, and Kaplan–Meier survival analysis. *Results:* GBM patients showed significantly higher levels of IL-22 compared to healthy controls (*p* = 0.001). ROC analysis demonstrated moderate diagnostic ability of IL-22 (AUC = 0.713), with high levels being a potential risk factor for the disease (OR= 2.51). A weak inverse correlation was found between IL-22 and neutrophil-to-lymphocyte ratio (NLR) (*p* = 0.048). Although IL-22 levels alone did not affect overall survival, patients with high levels of the cytokine and dense stromal infiltration of CD163+ macrophages tended to have shorter overall survival (*p* = 0.080). *Conclusions*: IL-22 is a potential diagnostic biomarker, probably reflecting the systemic inflammatory response in GBM. Its prognostic value might be contextually dependent on the tumor microenvironment, as high levels of IL-22 in combination with immunosuppressive macrophages may contribute to a more aggressive course of the disease.

## 1. Introduction

Glioblastoma multiforme (GBM) is the most frequent, most aggressive primary malignant tumor of the central nervous system in adults [1]. Despite advances in multimodal therapy, which includes maximal surgical resection, followed by radiotherapy and chemotherapy with temozolomide, the clinical outcome remains poor. GBM incidence varies in different populations: in developed countries, there are approximately 3–4 cases per 100,000 people yearly [2]. The median survival of patients is only about 14–18 months, and the 5-year survival rate is less than 5% [3]. The high invasiveness, genetic heterogeneity, and therapeutic resistance of GBM underscore the need to identify novel molecular biomarkers that could improve diagnosis and risk stratification.

Inflammation is a key component of the tumor microenvironment (TME) and has been recognized as a fundamental characteristic of cancers [4]. In GBM, the microenvironment is strongly immunosuppressive and highly infiltrated with tumor-associated macrophages and microglia (TAMs) [5]. These cells, particularly M2-type (pro-tumorigenic phenotype) expressing the membrane protein CD163, facilitate angiogenesis and immune evading [6]. In the TME, the communication between immune and tumor cells is mediated by a complex cytokine network. Interleukin-22 (IL-22), a member of the IL-10 superfamily of proteins, occupies a unique niche in this communication. Produced mainly by activated T-cells (Th22, Th17 and Th1), natural killer (NK) cells and innate lymphoid cells (ILCs), IL-22 does not act on immune cells but targets epithelial and stromal cells expressing the IL22R1/IL10R2 receptor complex [7,8,9]. Ligand binding to this receptor activates intracellular cascades including JAK1/Tyk2/STAT3 and PI3K/Akt, which are crucial for cell survival, proliferation, and tissue repair [9]. IL-22 itself is not considered a transforming oncogene. Experiments with transgenic mice show that its overexpression does not lead to the spontaneous occurrence of tumors [10]. However, IL-22 is defined as a tumor-promoting cytokine in a number of epithelial neoplasms, including lung, hepatocellular, pancreatic, colorectal, and gastric carcinoma, in which the presence of IL-22-producing cells and the expression of IL-22R1 on tumor cells stimulate tumor growth [11]. Also, experimental models of GBM suggest that IL-22 induces certain anti-apoptotic proteins (Bcl-2, Bcl-xL) and increases resistance to chemotherapy [12].

While experimental studies suggest a tumor-promoting role for IL-22, clinical evidence regarding circulating IL-22 levels in human GBM and their relationship with tumor-associated immune components remains limited and often contradictory. We therefore hypothesized that the increased infiltration of CD163+ TAMs in glioblastoma reflects a predominance of an M2-like immune phenotype and activation of immunosuppressive molecular pathways in the tumor microenvironment. Building on this, we sought to investigate whether this macrophage profile is associated with IL-22-dependent mechanisms that may contribute to tumor progression and poorer clinical outcomes. From a clinical perspective, the combined evaluation of IL-22 and CD163+ TAMs may provide a more comprehensive reflection of tumor–immune crosstalk by linking circulating inflammatory signals with local immune cell composition, thereby enhancing their potential prognostic relevance in glioblastoma.

We propose that exploring these interactions may provide meaningful insights into GBM immunobiology and outline directions for new therapeutic perspectives.

## 2. Materials and Methods

### 2.1. Study Subjects and Sample Collection

A total of 41 newly diagnosed with GBM patients were included in the present study. Patients were admitted to the University Hospital “Prof. Stoyan Kirkovich”, Stara Zagora, Bulgaria. All included subjects underwent surgical resection at the Department of Neurosurgery, and diagnosis was histologically confirmed at the Department of Clinical Pathology at the above-stated institution. Clinical and demographic parameters are described in Table 1.

The control group consisted of 46 clinically healthy volunteers recruited from the same region as the GBM patients. Individuals with a prior history of malignant disease, acute or chronic inflammatory conditions, autoimmune disorders, or current infections were excluded. Controls included 16 males (34.8%) and 30 females (65.2%). No formal matching for age or metabolic parameters was performed.

### 2.2. Quantification of Serum IL-22 Concentrations

Serum levels of IL-22 were quantified by a sandwich enzyme-linked immunosorbent assay (ELISA) using a commercially available kit (Shanghai Sunred Bio, Cat No 201-12-0039, Shanghai, China) according to the manufacturer’s instructions. Patient blood samples were collected prior to surgery. Approximately two milliliters of venous blood was collected in appropriate containers under standardized conditions, allowed to clot, and centrifuged to obtain serum, which was aliquoted and stored at −80 °C until analysis. Repeated freeze–thaw cycles were avoided. The samples from patients and controls were analyzed together at the same analytic badge to minimize inter-assay variability, and measurements were performed according to the manufacturer’s instructions. The results were calculated by reference to the standard curve constructed from the standards provided by the manufacturer, and results were expressed as pg/mL. Results were read on an EZ 400 Microplate Reader (Biochrom, Cambridge, UK) and calculated via Galapagos Software, v. 1.1a for EZ Readers.

### 2.3. Immunohistochemistry

Tissue specimens from patients were analyzed immunohistochemically. After fixation in 10% buffered formalin biopsies were embedded in paraffin and then cut to sections of 4 μm thickness. Next, they were dewaxed and endogenous peroxidase was blocked for 5 min with blocking reagent. Slides were washed 3 times with PBS and incubated with primary antibody for 1 h. After washing 3 times, the slides were incubated with marked polymer and then washed again. In the last phase, they were incubated with DAB substrate-chromogen and were washed again. At the end, they were contrastained with Mayer’s hematoxylin.

Mouse anti-human CD163 antibody (NCL-L-CD163, Leica Biosystems, Deer Park, IL, USA) in a dilution of 1:50, as well as the detection system EnVision™ FLEX+, Mouse, High pH, (Link) (K8002, DAKO, Glostrup, Denmark), was used for the immune reaction. Two qualified pathologists have scored the cases independently by counting the number of CD163+ TAMs in five separate microscopic fields of vision in both the tumor stroma and the tumor border. Discrepancies between observers were resolved by joint review to reach a consensus. CD163 positivity was defined based on specific cytoplasmic and membranous staining patterns, with appropriate internal controls used for comparison.

### 2.4. Statistical Analyses

Statistical analyses were performed using SPSS software, v.25 (IBM Corp., Armonk, NY, USA). Data distribution was assessed using the Kolmogorov–Smirnov test. For comparison of continuous variables between independent groups, the Mann–Whitney U test was applied, and for paired samples the Wilcoxon signed-rank test was used. Correlations between continuous variables were assessed using Spearman’s rank correlation coefficient (ρ). Receiver operating characteristic (ROC) curve analysis was conducted to evaluate the discriminatory ability of serum IL-22 levels for differentiating GBM cases from controls. The area under the ROC curve (AUC) with standard error (SE) and 95% confidence intervals (CIs) was calculated. Comparisons between ROC curves were performed using the DeLong test.

Binary logistic regression analysis was applied to assess the independent association between serum IL-22 levels and GBM risk. IL-22 concentrations were log-transformed prior to inclusion in regression models to achieve approximate normality. Age and sex were included as covariates. Model fit was evaluated using the omnibus χ^2^ test, Nagelkerke R^2^, and overall classification accuracy. Results are presented as regression coefficients (B), odds ratios (ORs), and 95% confidence intervals.

Survival analyses were performed using Kaplan–Meier curves, and differences between survival distributions were assessed with the log-rank test.

All statistical tests were two-tailed, and a *p*-value < 0.05 was considered statistically significant. Results with *p*-values between 0.05 and 0.10 were interpreted as indicative of a statistical tendency.

## 3. Results

**Figure 1:**
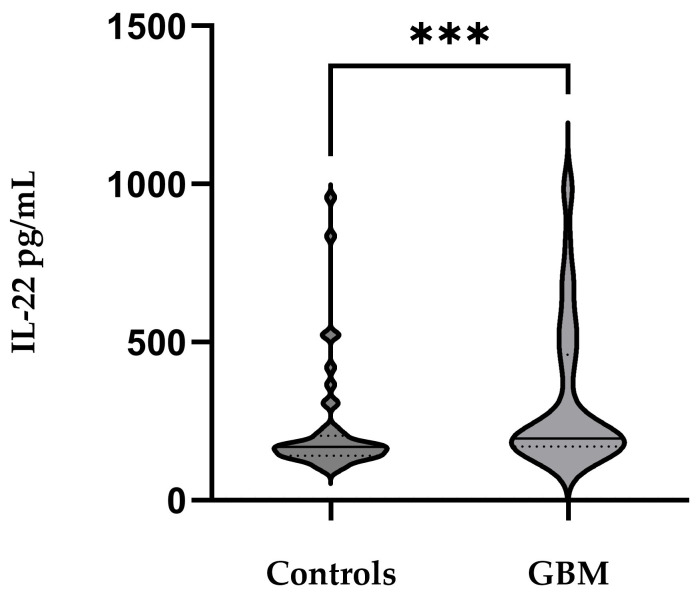
Serum IL-22 levels are significantly elevated in patients with glioblastoma compared with healthy controls. Horizontal lines indicate median values, Mann–Whitney U = 527.0, *** *p* = 0.001.

A Mann–Whitney *U* test revealed significantly higher IL-22 levels in GBM cases compared to controls (U = 527.0, Z = −3.26, *p* = 0.001) with mean levels of 321.94 ± 237.39 pg/mL and 222.74 ± 173.96 pg/mL, respectively (Figure 1). Approximately equal sera IL-22 was detected among male (323.58 ± 239.40 pg/mL) and female (318.43 ± 253.64) GBM patients, *p* = 0.679.

**Figure 2:**
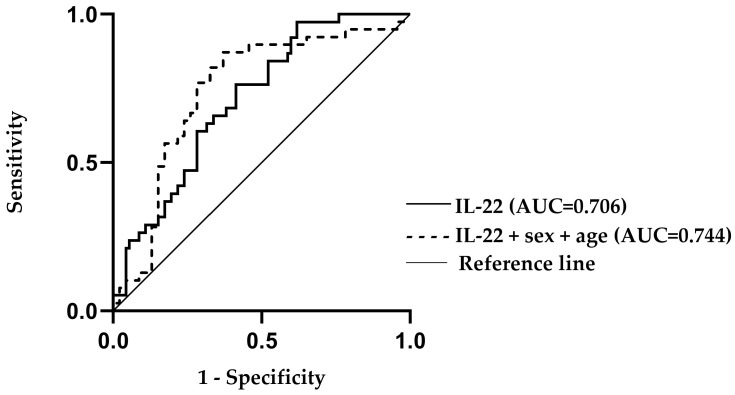
Receiver operating characteristic (ROC) analysis demonstrating the moderate discriminatory ability of serum IL-22 levels for distinguishing glioblastoma patients from healthy controls (*p* < 0.01). The diagnostic performance is shown for IL-22 alone (AUC = 0.706) and for IL-22 adjusted for age and sex (AUC = 0.744), indicating a modest improvement when demographic covariates are included.

Receiver operating characteristic (ROC) analysis demonstrated that serum IL-22 levels moderately discriminated GBM cases from controls (AUC = 0.713, SE = 0.055, 95% CI: 0.606–0.820, *p* < 0.001). Model performance improved when age and sex were added as covariates (AUC = 0.744, SE = 0.056, 95% CI: 0.633–0.854, *p* < 0.001), although the increase in AUC did not reach statistical significance (*p* = 0.620), Figure 2.

**Figure 3:**
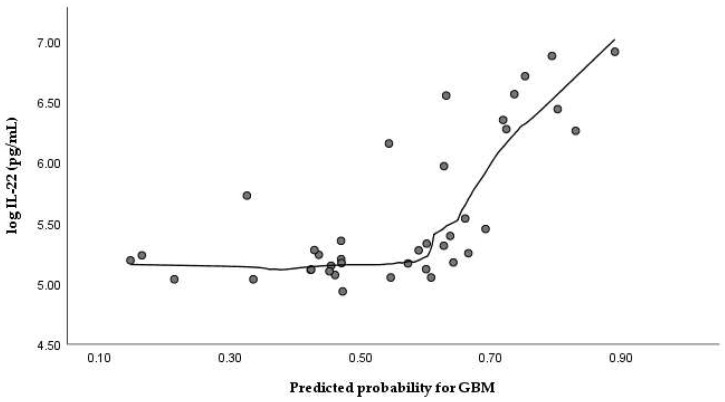
Relationship between serum IL-22 levels and model-derived predicted probability of GBM based on multivariable logistic regression, illustrating the contribution of IL-22 to risk estimation at the individual level. Points represent individual participants.

A multivariable binary logistic regression model including log-transformed IL-22 concentrations, age, and sex demonstrated a statistically significant overall fit (χ^2^ = 15.559, df = 3, *p* = 0.001), with a Nagelkerke R^2^ of 0.224 and an overall classification rate of 68.2%. Higher log-IL-22 levels were independently associated with increased odds of GBM (B = 0.920 ± 0.456, *p* = 0.044), corresponding to an odds ratio (OR) of 2.51 (95% CI: 1.03–6.15), Figure 3. Expectedly, age was also an independent predictor (B = 0.032 ± 0.015, *p* = 0.038; OR = 1.03, 95% CI: 1.00–1.06). Sex did not reach statistical significance in the multivariable model (B = 0.774 ± 0.481, *p* = 0.108; OR = 2.17, 95% CI: 0.85–5.54), indicating that the association between IL-22 and GBM was not sex-dependent after adjustment for age; see Table 2.

**Figure 4:**
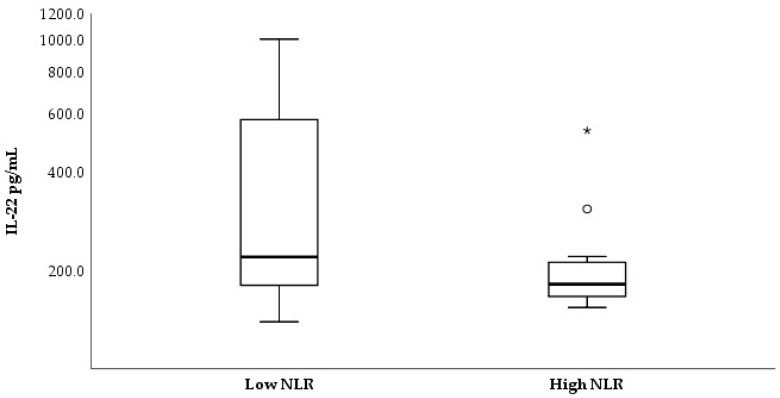
Serum IL-22 levels according to neutrophil-to-lymphocyte ratio (NLR) status in GBM patients. Patients were stratified into low- and high-NLR groups based on the cohort median value (NLR = 4.01). A tendency toward higher IL-22 levels was observed in the low-NLR group (Mann–Whitney U = 95.5, *p* = 0.058). Circle (◦) indicate outlier and asterisks (*) indicate extreme outlier.

**Figure 5:**
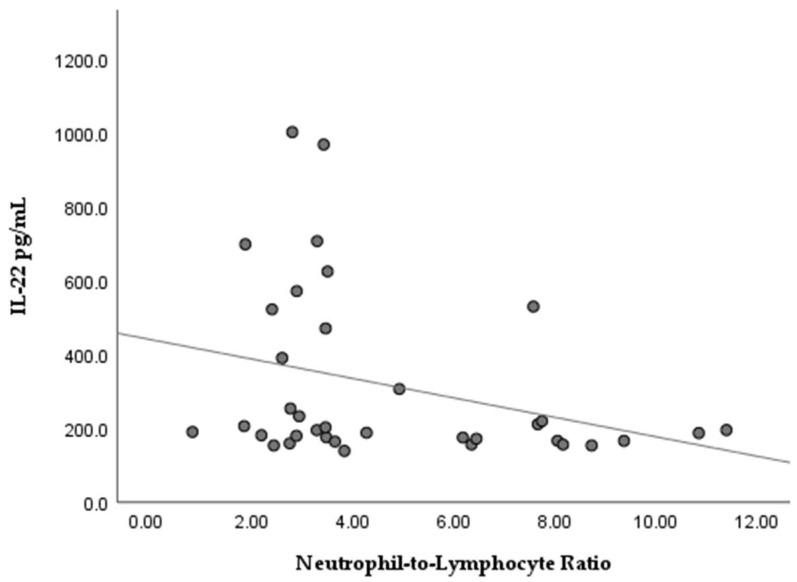
Relationship between serum IL-22 concentrations and NLR in GBM patients, showing a weak inverse association (Spearman’s ρ = −0.331, *p* = 0.048).

Non-parametric analyses of serum IL-22 concentrations with regard to clinical data of the studied subjects were applied. The GBM group was dichotomized to low/high groups according to the median values for the neutrophil-to-lymphocyte ratio (NLR, median cut-off = 4.01) and fasting pre-operative glucose levels (median cut-off = 6.74 mmol/L). As shown in Figure 4, stratification by median NLR revealed a tendency toward higher serum IL-22 levels in patients with low NLR (Mann–Whitney U = 95.5, *p* = 0.058). Spearman rank analysis further identified a modest inverse association between continuous NLR and IL-22 concentrations (ρ = −0.331, *p* = 0.048; Figure 5).

**Figure 6:**
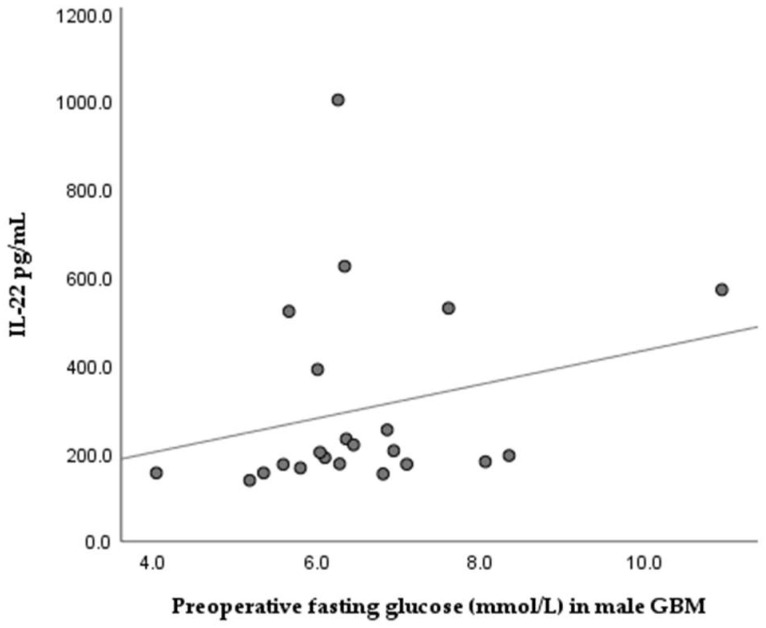
Relationship between serum IL-22 levels and pre-operative fasting glucose concentrations in male GBM patients, showing a tendency toward a positive association (Spearman’s ρ = 0.397, *p* = 0.067). No significant correlations were observed between serum IL-22 and CRP levels either in the whole group or in the sex-stratified GBM group. Also, there was no statistically significant difference in the overall survival time of GBM cases according to IL-22 serum levels.

We found no significant difference between serum IL-22 and preoperative fasting glucose levels in GBM patients (Mann–Whitney U = 171.00, *p* = 0.792). When we analyzed the GBM groups according to sex, we detected a tendency towards positive correlation between serum IL-22 concentration and glucose levels in males (Spearman rho = 0.397, *p* = 0.067); see Figure 6.

**Figure 7:**
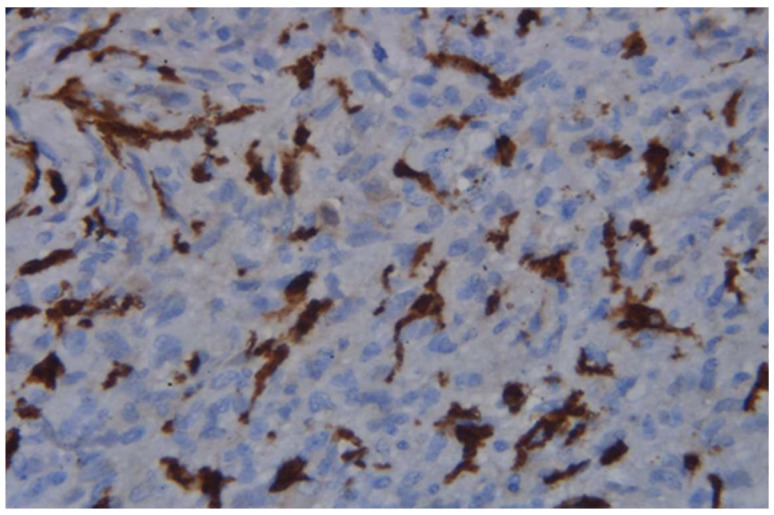
CD163-positive tumor-associated macrophages in the tumor stroma of GBM biopsy, ×400, scale bar = 20 µm.

**Figure 8:**
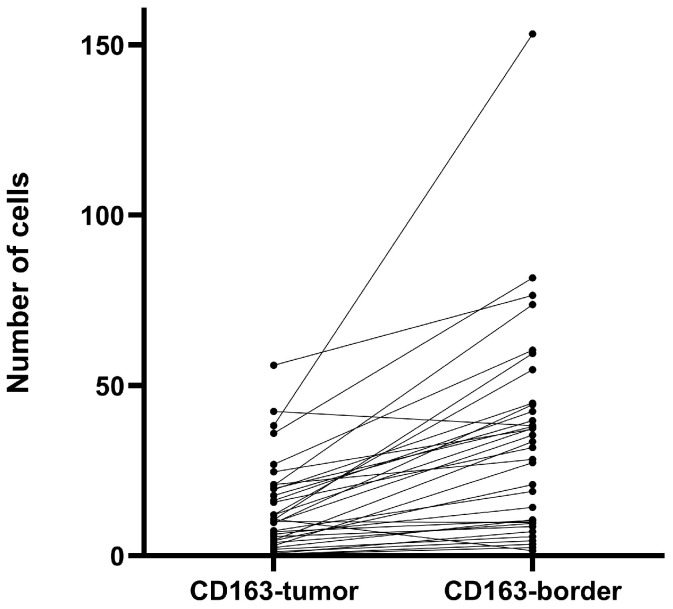
Paired analysis of CD163+ macrophage density at the tumor border compared with the tumor stroma in GBM (Wilcoxon signed-rank test, *p* < 0.001). Each line represents a paired sample from the same patient. Although no significant differences in IL-22 levels were observed between patients stratified by median CD163 expression in the tumor stroma or tumor border (Mann–Whitney U test, *p* > 0.1), cases with low infiltration of CD163+ cells had increased levels of serum IL-22. Therefore, subsequent analyses were focused on the subgroup of patients with high serum IL-22 levels (classified according to the estimated median) in order to explore whether local CD163+ macrophage density further stratified clinical and demographic data within a systemically inflamed context. Among patients with high serum IL-22 levels, a tendency toward shorter overall survival was observed in cases with higher CD163+ macrophage density in the tumor stroma compared with those with lower CD163 expression (log-rank test, *p* = 0.080); see Figure 9.

Evaluation of CD163+ cell density (Figure 7) in tumor biopsy specimens from the studied GBM cases demonstrated significantly higher infiltration at the tumor border compared with the tumor stroma (Wilcoxon signed-rank test, Z = −4.69, *p* < 0.001, r ≈ 0.80), Figure 8.

**Figure 9:**
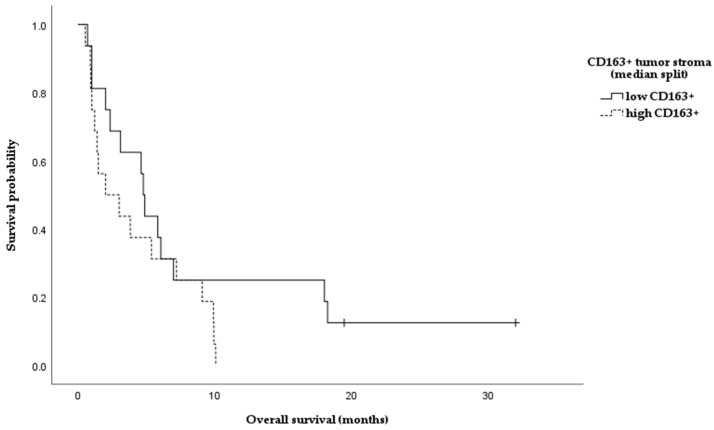
Kaplan–Meier overall survival curves stratified by low versus high CD163⁺ macrophage infiltration in the tumor stroma of GBM patients. A tendency toward shorter survival was observed in cases with high CD163+ infiltration (log-rank test, *p* = 0.080).

Also, in female patients with high serum IL-22 levels, shorter overall survival was observed in cases with higher CD163+ macrophage density in the tumor stroma compared with those with lower CD163 expression (log-rank test, *p* = 0.008); however, this finding was based on a small number of cases in the subgroup.

## 4. Discussion

### 4.1. Serum IL-22 in GBM

IL-22, produced mainly by activated T-lymphocytes (Th17, Th22), belongs to the IL-10 superfamily of proteins and engages the IL-22R1/IL-10R2 complex, stimulating the JAK/STAT3 and PI3K/Akt signal pathways [13]. In the context of tumorigenesis, IL-22 induces proliferative and anti-apoptotic signaling in target cells, which are predominantly non-myeloid. [14]. While IL-22 normally contributes to tissue repair, tumor cells may exploit its activity to support their own growth and survival. Consistently, experimental data reported that IL-22 supports GBM cell survival and proliferation, activating the STAT3/PIK3-Akt cascades [15]. Besides its direct effect on tumor cells, IL-22 also modulates anti-tumor immunity; for example, increased IL-22 was associated with impaired NK-cell function, resulting in tumor immune evasion [14].

The results from our study demonstrated that serum IL-22 levels were significantly higher in GBM patients compared to healthy control individuals (*p* = 0.001), which confirms that in GBM, the systemic inflammatory response involves IL-22. This observation is consistent with data showing that a number of malignant tumors (lung, liver, stomach, colon, pancreas, etc.) develop with elevated levels of IL-22 in tumor tissue and systemic circulation [16]. ROC analyses in our study showed a moderate discriminatory ability of IL-22 for GBM (AUC = 0.713). Although far from an ideal biomarker, IL-22 indicated potential to complement the diagnostic panel of GBM. The addition of age and sex as covariates slightly improved the model (AUC = 0.744), suggesting a potential diagnostic value of IL-22 when considered alongside age and sex. It should be noted that residual confounding related to age, sex, or metabolic status cannot be entirely excluded and may have contributed to the observed diagnostic performance of IL-22. Therefore, these findings should be interpreted within the context of the study design and cohort characteristics. Additionally, we found an association between higher serum IL-22 levels and increased odds of GBM (OR = 2.5, *p* = 0.044), suggesting that IL-22 may emerge as a candidate biomarker that requires further validation in larger, independent cohorts. These observations are in accordance with numerous studies in other carcinomas suggesting that the deregulation of the IL-22/IL22-R axis contributes to carcinogenesis [17]. In lung cancer, it has been shown that increased expression of IL-22R1 in tumor cells correlates with shorter survival time [18], and upregulated IL-22 expression in laryngeal tumor tissue has been associated with a more aggressive phenotype [19].

An interesting observation in our study was a modest inverse association between serum IL-22 levels and the systemic inflammatory marker NLR (ρ = −0.331, *p* = 0.048). Also, the stratification of the GBM group according to the median revealed a tendency for higher IL-22 levels in GBM cases with low NLR. Normally, high NLR indicates systemic inflammation combined with immunosuppression, which is a basis for worse prognosis in a number of cancers, including GBM [20,21], Thus, our observations seem contrary given the expected pro-inflammatory role of IL-22. One possible explanation could be that elevated serum IL-22 reflects the adaptive immune responses mediated by Th22 and Th17 cells, which are abundant in patients with lower neutrophil inflammation. In other words, patients with lower NLR could have more active Th-cells, which are the primary source of IL-22. In line with this, Kargl et al. [21] reported that in non-small cell lung cancer, high neutrophil counts were significantly associated with lower Th17 and Th1 T-cell infiltrates, whereas cases with lower neutrophil prevalence showed greater Th17 (and Th1) presence [22]. Similar findings were reported for atopic dermatitis, which is a chronic inflammatory disease with a shift in the immune profile towards Th-22 combined with low neutrophil content [23]. With regard to metabolic factors, our study did not find a significant correlation between serum IL-22 levels and pre-operative fasting glucose (ρ = 0.790). In male GBM cases, we detected a tendency for positive correlation (ρ = 0.397, *p* = 0.067), i.e., slightly higher serum IL-22 in GBM males with increased glucose levels, but without statistical significance. Despite the lack of association in our study, clinical data suggest that systemic hyperglycemia might aggravate GBM outcome [24,25]. It is supposed that hyperglycemia accelerates tumor metabolism (the Warburg effect) and provokes a pro-inflammatory environment, supporting tumor growth.

### 4.2. IL-22 and the Tumor Microenvironment

Important components of the tumor microenvironment in GBM include tumor-associated macrophages (TAMs). As a membrane marker, CD163 is characteristic of alternatively activated (M2) macrophages that produce pro-inflammatory mediators (e.g., IL-10), supporting tumor growth [15,26]. Our histologic findings confirm a significant infiltration of GBM tissue with CD163+. Interestingly, the infiltration was more pronounced in the invasive border of the tumor rather than the stroma (*p* < 0.001), corresponding to the assumption that macrophages accumulate at the border between tumor and healthy brain tissue. Probably this infiltration pattern supports tissue invasion rather than mounting an effective anti-tumor response. In our cohort, we did not find any significant associations between serum IL-22 and CD163+ cells. However, in GBM cases with elevated serum IL-22, increased CD163+ macrophage infiltration in the tumor stroma was associated with shorter overall survival (log-rank, *p* = 0.08). This observation was more clear when we stratified the patients by sex: women had significantly shorter survival compared to men (log-rank, *p* = 0.008), although the small number of cases in the subgroup should be considered. It is possible that the immune response in different genders modulates the effects of IL-22. Sex differences in immunity have been described, with women often exhibiting stronger humoral and Th17/Th2 responses, while men exhibit stronger Th1 responses [27]. Some studies have shown a higher frequency of IL-17/IL-22-producing T cells in women compared to men, which may indicate that women’s immune systems are more prone to secreting cytokines such as IL-22. If women with GBM generate a more robust IL-22-mediated inflammatory response, it could recruit and activate more immunosuppressive macrophages into the tumor. As a result, the combination of high IL-22 and M2 infiltrate may disproportionately be detrimental to women, explaining the observed lower survival in this subgroup. Although this explanation is hypothetical, it is supported by the concept that sex influences tumor immunology and may modulate the success of various therapeutic approaches (including immune therapies). However, further studies with larger numbers of patients are needed to confirm these sex-specific interactions between IL-22, macrophages, and prognosis in GBM. In line with our preliminary observations, it is plausible to suggest that IL-22 and TAMs act synergistically in supporting tumor development. It is well established that M2-polarized macrophages (CD163+, CD206+) inhibit the anti-tumor T-cell response, stimulate angiogenesis and tissue remodeling, and thus facilitate GBM progression. Lui et al. [26] demonstrated that higher density of these macrophages correlates with shorter overall survival. On the other hand, IL-22 may contribute to maintaining this immunosuppressive setting. It has been experimentally shown that IL-22 directly promotes polarization of monocytes towards the M2-phenotype, enhancing the expression of Arg1, CD163, and IL-10 through the STAT3 signal pathway [15]. Consequently, in patients with high systemic IL-22, the immune landscape could easily shift towards M2-dominant tumor infiltration, worsening the immune control over the tumor.

### 4.3. Diagnostic and Prognostic Potential of IL-22 in GBM

Our results and contextual analysis suggest that IL-22 deserves attention as a biomarker in GBM. Diagnostically, the moderate sensitivity and specificity (AUC ~0.71) indicate that IL-22 alone is not sufficient for screening or a definitive diagnosis but could be when combined with other markers to improve accuracy. In this context, previous studies have shown that integrating inflammatory cytokines and markers such as NLR can enhance diagnostic and prognostic stratification in GBM, and IL-22 may represent one component of such an immunological signature [21]. With respect to risk associations, the observed odds ratios in our cohort (Table 2) reflect potential relationship derived from a case–control analysis rather than true predictive performance in a prospective clinical setting and should be interpreted accordingly. In terms of prognosis, serum IL-22 levels alone were not significantly associated with overall survival in our cohort. This distinguishes IL-22 from other cytokines such as IL-6 and IL-8, which have been more consistently associated with disease aggressiveness [28]. However, our data suggest that the prognostic relevance of IL-22 may be context-dependent, manifesting only when considered in combination with tumor microenvironment characteristics, specifically, CD163+ infiltration. In the presence of an immunosuppressive surrounding, IL-22 may contribute to tumor-promoting processes such as proliferation, migration, and immune evasion. Similar context-dependent interactions between circulating cytokines and local immune components have been reported in other malignancies, including non-small cell lung carcinoma and colorectal cancer [29,30]. Taken together, these findings support the concept of IL-22 as a dual player—serving as a possible marker of immune activation while potentially contributing to tumor progression under specific conditions—while highlighting the need for validation in larger, prospective studies. The present study has several limitations that should be considered when interpreting the results. First, given the preliminary observational design and the lack of formal power calculations, the available sample size may not have been sufficient to detect weaker associations, particularly in subgroup and sex-stratified analyses. Next, while our methodology provides relevant exploratory insights, functional assays to dissect the mechanistic role of IL-22 and CD163+ macrophages in GBM progression were not available and should be addressed in future studies. Also, the use of serum IL-22 levels as a systemic marker does not allow for direct discrimination of local tumor effects from systemic immune processes. Similarly, the assessment of CD163+ infiltration provides a quantitative but not a functional characterization of the macrophage phenotype. Future studies combining serum, tissue, and functional analyses would contribute to a deeper understanding of the role of IL-22 and CD163+ in glioblastoma.

## 5. Conclusions

In conclusion, the role of IL-22 in Glioblastoma multiforme is expressed in complex interactions between the systemic inflammatory response and the tumor microenvironment. Elevated serum levels of IL-22 differentiate diseased from healthy individuals and could suggest active tumor-associated immunopathology. Accumulating evidence of IL-22 as an inflammatory mediator and biomarker in oncology supports the idea that it is a potential target for diagnosis and therapy. Our study contributes to this picture by highlighting the importance of IL-22 in relation to the patient’s immune status and tumor microenvironment and encourages further investigation into IL-22 in glial tumors.

## Figures and Tables

**Table 1:** Clinical and demographic parameters for GBM patients.

Parameter	Category	Number (%)
Age	Mean ± SD	62.74 ± 13.67
Gender	Male Female	23 (56%) 18 (44%)
Tumor number	Solitary Multiple	26 (63%) 15 (37%)
Tumor occurrence	Primary Recurrence	37 (90%) 4 (10%)
Type of surgery	GTR (gross total resection) STR (subtotal resection)	23 (56%) 18 (44%)
Frontal lobe involvement	Yes No	16 (39%) 25 (61%)
Laterality	Left Right	25 (61%) 16 (39%)
Fasting glucose	Median (mmol/L)	6.74
NLR	Median	4.01
Survival status at the end of follow-up	Deceased Survived	39 (95%) 2 (5%)
Overall survival time	Mean ± SD (months)	6.87 ± 7.92

**Table 2:** Multivariable binary logistic regression analysis evaluating log-IL-22, age, and sex as predictors of GBM.

Variable	B	SE	Wald	*p*-Value	OR	95% CI for OR
IL-22 (log)	0.920	0.456	4.066	0.044	2.509	1.03–6.15
Age (years)	0.032	0.015	4.285	0.038	1.032	1.00–1.06
Male (vs. female)	0.774	0.481	2.587	0.108	2.168	0.85–5.54
Constant	−7.324	2.574	8.096	0.004	0.001	

Model statistics: χ^2^(3) = 15.559, *p* = 0.001; −2 Log likelihood = 101.699; Nagelkerke R^2^ = 0.224; overall classification accuracy = 68.2%.

## Data Availability

The original contributions presented in this study are included in the article. Further inquiries can be directed to the corresponding author.

## References

[1] Price M., Ballard C., Benedetti J., Neff C., Cioffi G., Waite K.A., Kruchko C., Barnholtz-Sloan J.S., Ostrom Q.T. (2024). CBTRUS Statistical Report: Primary Brain and Other Central Nervous System Tumors Diagnosed in the United States in 2017–2021. Neuro. Oncol..

[2] Królikowska K., Błaszczak K., Ławicki S., Zajkowska M., Gudowska-Sawczuk M. (2025). Glioblastoma—A Contemporary Overview of Epidemiology, Classification, Pathogenesis, Diagnosis, and Treatment: A Review Article. Int. J. Mol. Sci..

[3] Sipos D., Raposa B.L., Freihat O., Simon M., Mekis N., Cornacchione P., Kovács Á. (2025). Glioblastoma: Clinical Presentation, Multidisciplinary Management, and Long-Term Outcomes. Cancers.

[4] Hanahan D., Weinberg R.A. (2011). Hallmarks of cancer: The next generation. Cell.

[5] Hambardzumyan D., Gutmann D.H., Kettenmann H. (2016). The role of microglia and macrophages in glioma maintenance and progression. Nat. Neurosci..

[6] Mantovani A., Marchesi F., Malesci A., Laghi L., Allavena P. (2017). Tumour-associated macrophages as treatment targets in oncology. Nat. Rev. Clin. Oncol..

[7] Dudakov J.A., Hanash A.M., van den Brink M.R.M. (2015). Interleukin-22: Immunobiology and pathology. Annu. Rev. Immunol..

[8] Ouyang W., O’Garra A. (2019). IL-10 Family Cytokines IL-10 and IL-22: From Basic Science to Clinical Translation. Immunity.

[9] Jiang R., Sun B., Birbrair A. (2021). IL-22 Signaling in the Tumor Microenvironment. Tumor Microenvironment Cham: Springer International Publishing.

[10] Markota A., Endres S., Kobold S. (2018). Targeting interleukin-22 for cancer therapy. Hum. Vaccin. Immunother..

[11] Sabat R., Ouyang W., Wolk K. (2014). Therapeutic opportunities of the IL-22-IL-22R1 system. Nat. Rev. Drug Discov..

[12] Lim C., Savan R. (2014). The role of the IL-22/IL-22R1 axis in cancer. Cytokine Growth Factor Rev..

[13] Akil H., Abbaci A., Lalloué F., Bessette B., Costes L.M.M., Domballe L., Charreau S., Guilloteau K., Karayan-Tapon L., Bernard F.-X. (2015). IL22/IL-22R Pathway Induces Cell Survival in Human Glioblastoma Cells. PLoS ONE.

[14] Luo W., Yu B., Qin Q.-Y., Lu J.-M., Qin S.-Y., Jiang H.-X. (2016). Interleukin-22 promotes macrophage M2 polarization via STAT3 pathway. Int. J. Clin. Exp. Med..

[15] Weinberg F.D., Ramnath N. (2018). Targeting IL22: A potential therapeutic approach for Kras mutant lung cancer?. Transl. Lung Cancer Res..

[16] Hernandez P., Gronke K., Diefenbach A. (2018). A catch-22: Interleukin-22 and cancer. Eur. J. Immunol..

[17] Bi Y., Cao J., Jin S., Lv L., Qi L., Liu F., Geng J., Yu Y. (2016). Interleukin-22 promotes lung cancer cell proliferation and migration via the IL-22R1/STAT3 and IL-22R1/AKT signaling pathways. Mol. Cell. Biochem..

[18] Ji W., Li J., Wang X., Gao D., Zhang T. (2021). Increased expression of interleukin-22 and its receptor is relevant to poor prognosis in laryngeal squamous cell carcinoma: A case control trial. Medicine.

[19] Zemskova O., Yu N.Y., Löser A., Leppert J., Rades D. (2024). Prognostic Role of Platelet-to-Lymphocyte and Neutrophil-to-Lymphocyte Ratios in Patients Irradiated for Glioblastoma Multiforme. Cancer Diagn. Progn..

[20] Jarmuzek P., Kozlowska K., Defort P., Kot M., Zembron-Lacny A. (2023). Prognostic Values of Systemic Inflammatory Immunological Markers in Glioblastoma: A Systematic Review and Meta-Analysis. Cancers.

[21] Kargl J., Busch S.E., Yang G.H.Y., Kim K.H., Hanke M.L., Metz H.E., Hubbard J.J., Lee S.M., Madtes D.K., McIntosh M.W. (2017). Neutrophils dominate the immune cell composition in non-small cell lung cancer. Nat. Commun..

[22] Dhingra N., Suárez-Fariñas M., Fuentes-Duculan J., Gittler J.K., Shemer A., Raz A., Fischetti V.A., Krueger J.G., Guttman-Yassky E. (2013). Attenuated neutrophil axis in atopic dermatitis compared to psoriasis reflects TH17 pathway differences between these diseases. J. Allergy Clin. Immunol..

[23] Lu V.M., Goyal A., Vaughan L.S., McDonald K.L. (2018). The impact of hyperglycemia on survival in glioblastoma: A systematic review and meta-analysis. Clin. Neurol. Neurosurg..

[24] De S., Banerjee S., Dey G., Banerjee S., Kumar S.K.A. (2025). Interplay Between Diabetes, Obesity and Glioblastoma Multiforme, and the Role of Nanotechnology in Its Treatment. J. Nanotheranostics.

[25] Liu L., Wang R., Alifu A., Xiao Y., Liu Y., Qian C., Zhao M., Tang X., Xie Y., Shi Y. (2024). Hypoxia-driven M2-polarized macrophages facilitate the epithelial-mesenchymal transition of glioblastoma via extracellular vesicles. Theranostics.

[26] Bernardi S., Toffoli B., Tonon F., Francica M., Campagnolo E., Ferretti T., Comar S., Giudici F., Stenner E., Fabris B. (2020). Sex Differences in Proatherogenic Cytokine Levels. Int. J. Mol. Sci..

[27] Zhu V.F., Yang J., LeBrun D.G., Li M. (2012). Understanding the role of cytokines in Glioblastoma Multiforme pathogenesis. Cancer Lett..

[28] Xu L., Cao P., Wang J., Zhang P., Hu S., Cheng C., Wang H. (2024). IL-22: A key inflammatory mediator as a biomarker and potential therapeutic target for lung cancer. Heliyon.

[29] Koltsova E.K., Grivennikov S.I. (2014). IL-22 Gets to the Stem of Colorectal Cancer. Immunity.

[30] Ruan H.X., Fang Y.L., Qin X.N., Lin L. (2025). Interleukin-22 promotes cancer stemness and chemotherapy resistance in colorectal cancer via epidermal growth factor receptor/extracellular signal-regulated kinase pathway. World J. Gastrointest. Oncol..

[31] (2025). 37th European Congress of Pathology—Abstracts. Virchows Arch..

